# Emotion regulation profiles of development of depressive symptomatology: a longitudinal study.

**DOI:** 10.1093/geroni/igab046.3116

**Published:** 2021-12-17

**Authors:** Teresa Paniagua, Virginia Fernández-Fernández, MªÁngeles Molina Martínez

**Affiliations:** 1 Universidad Europea de MAdrid, Majadahonda, Madrid, Spain; 2 Universidad Nacional de Educación a Distancia, Madrid, Madrid, Spain; 3 Universidad Francisco de Vitoria, Madrid, Madrid, Spain

## Abstract

Introduction:COVID-19 pandemic has had a psychological impact on the eldest population. The aim is to analyse whether there are differences depending on the emotional regulation profile shown by a group of older people 6 months before the pandemic and the depressive symptomatology of these people at the same time, during home confinement and 8 months later. Method:Longitudinal study, sample of people over 65, three evaluation measures: WAVE1 (6 months before COVID-19,N=305;M=73.63;58.9% women), WAVE2 (house confinement;N=151;M=73.14;59.6% women) and WAVE3 (8 months later;N=91;M=72.62;64.70% women). We measured depressive symptomatology (CES-D; Radloff, 1977) and nine emotional regulation strategies (CERQ-S; Garnefski et al., 2001; Carvajal et al., 2020), with which 3 clusters were preset (after dendogram inspection and K means analysis). Three mean difference analyses (one-factor ANOVA) were performed taking as factor profiles and as outcomes variables depression in each wave. Results:profile 1, people use adaptive cognitive-emotional regulation strategies; profile 2, those with low levels of strategies (adaptive and maladaptive); profile 3, high scores in maladaptive strategies. Statistically significant differences between profiles 1 and 3, in the pre-confinement depression variable (F'2,91=6.18;p=.00) and during confinement (F'2,91=4.02;p=.02). Profile 3 higher depressive symptomatology (x̄1=17.16;x̄2=16.80) than 1 (x̄1=8.41;x̄2=9.65). Differences between profile 1 and 2 and 3 in depression 8 months after confinement (F’2,91=4.02;p=.02). Profile 1 lower levels of depression (x̄3=98.00) than 2 (x̄3=15.78) and 3 (x̄3=14.20). Profiles explain 12.3%, 8.4% and 12.5% of the depression variance in each wave. Conclusions:a "protected profile" (1), a "medium-term vulnerable profile" (2) and a "vulnerable profile" (3) to the development of depressive symptomatology.

